# Single Cell Protein Production From Ethanol: Model‐Based Bioreactor Operation at Industrial Scale

**DOI:** 10.1002/bit.28969

**Published:** 2025-03-21

**Authors:** Eduardo Almeida Benalcázar, Wouter A. van Winden, Lars Puiman, John A. Posada, Mickel L. A. Jansen, Henk Noorman, Adrie J. J. Straathof

**Affiliations:** ^1^ Department of Biotechnology Delft University of Technology Delft the Netherlands; ^2^ dsm‐firmenich, Center for Bioprocess Innovation Delft the Netherlands

**Keywords:** bioreactor modeling, characteristic times, heat transfer, O_2_ transfer, single cell protein, technical feasibility

## Abstract

Alternative fermentation feedstocks such as ethanol can be produced from CO_2_ via electrocatalytic processes that coproduce O_2_. In this study, industrial‐scale fermentation of ethanol with pure O_2_ for single cell protein (SCP) production was studied using a modeling approach. This approach considered (i) microbial kinetics, (ii) gas–liquid transfer, and (iii) an exploration of potential operational constraints. The technical feasibility for producing up to 58 kt/y of SCP in a 600 m^3^ bubble column operating in continuous mode was assessed and attributed mainly to a high O_2_ transfer rate of 1.1 mol/(kg h) through the use of pure O_2_. However, most of the pure O_2_ fed to the fermenter remains unconsumed due to the large gas flows needed to maximize mass transfer. In addition, biomass production may be hampered by high dissolved CO_2_ concentrations and by large heat production. The model estimates a microbial biomass concentration of 114 g/kg, with a yield on ethanol of 0.61 g_x_/g_ethanol_ (> 95% $Y_{x/s}^{\max}$). Although the large predicted O_2_ transfer capacity seems technically feasible, it needs further experimental validation. The model structure allows the analysis of alternative substrates in the same way as identifying the best carbon feedstock.

## 1 Introduction

Protein intake has increased with the global average income per capita, currently reaching an average of 82 g per day (Andreoli et al. 2021; FAO 2023). Most of the proteins are sourced from crops such as soybeans, lentils, and chickpeas, but the fraction of protein originating from animal sources has increased to 38% (FAO 2023). Considering that red meat production contributes about 50% of the total greenhouse gas emissions derived from food production and requires extensive amounts of land for grazing or feed crops cultivation (Godfray et al. 2018), the environmental burden of protein production could then bring the United Nations Sustainable Development Goals number 2 (zero hunger) into conflict with numbers 13 (climate action) and 15 (life on land), as the human population and middle class grow.

In response, the development of alternative proteins (e.g., single cell microbial protein [single cell protein, SCP], insects, algae, cultured meat [Godfray et al. 2018], and specific proteins produced by microbes [Ritala et al. 2017]) for human diets is gaining attention due to the possible decoupling of their production systems from traditional agriculture (Leger et al. 2021). Industrial production of edible protein‐rich microbial cells appeared in the late 1960s. SCP has been used mostly as animal feed, but also as food after lengthy regulatory processes for governmental approval (Ritala et al. 2017; Tannenbaum and Wang 1977; Whittaker et al. 2020). By 2016, 43 companies were active in SCP production from carbohydrates, *n*‐alkanes, methanol, and ethanol using fungi (including yeasts) and bacteria and from CO_2_ plus energy from light using phototrophs or energy from knallgas (mixtures of H_2_ and O_2_) using bacteria (Nyyssölä et al. 2022; Ritala et al. 2017).

As an example, one can consider industrial SCP production from glucose. A 155 m^3^ bioreactor was used through the 1980s and possibly early 1990s by Marlow Foods to produce *Quorn*, SCP approved for human consumption. The bioreactor was a 50‐m‐tall airlift with an external loop operating as a chemostat at 30°C and pH 6 (Moore et al. 2020; Trinci 1991; Trinci 1992; Wiebe 2002). The microorganism in this system was the filamentous fungus *Fusarium venenatum*, with a substrate affinity constant of 0.03 mmol/kg (Wiebe et al. 1992). Air and ammonia were both sparged at the bottom of the riser; pure O_2_ was injected at the entrance of the downcomer to avoid anaerobic metabolism; and the substrate was fed at the exit of the downcomer, where an internal cooling coil was installed (Trinci 1991; Trinci 1992).

Seeking to improve the efficiency of energy, water, and land use for protein production from agriculturally produced glucose, electrocatalytic reduction of CO_2_ and/or water with renewable electricity may be used for renewable production of C_1_–C_4_ platform compounds, directly or indirectly, such as methane, CO, methanol, ethanol, isopropanol, 2,3‐butanediol, formic acid, oxalic acid, acetic acid, and propionic acid (Cabau‐Peinado et al. 2024; Chaitanya et al. 2023; Fackler et al. 2021; 2021; Huang et al. 2021; Liew et al. 2022; Mikulčić et al. 2019). The production of these carbon/electron carriers leads to the concomitant production of O_2_, which may be used for enhancing productivity in protein fermentation. The O_2_ may also be used, within integrated industrial complexes, to enhance protein production from other waste substrates, such as those containing glucose, xylose, or glycerol (Jones et al. 2020; Maza et al. 2024). Table 1 shows a list of substrates potentially useful for SCP production.

**Table 1:** Substances that can potentially be used for SCP production.

Substance	Degree of reduction (mol_e‐_/mol)	State at 25°C^a^	Solubility in water at 25°C (g/kg)^a^	Price		Category
				€/kg, [€/kmol_e‐_]^c^	Source^h^	
Glucose	24	Solid	909	0.30, [2.25]^d^	A	A
Glycerol	14	Liquid	Miscible	0.25, [1.64]^d^	B	A
Methanol	6	Liquid	Miscible	0.40, [2.14]^e^	C	A
Ethanol	12	Liquid	Miscible	0.60, [2.30]^d^	A	A
Methane	8	Gas	2.52 × 10^−2^ b	0.60, [1.20]^d^,^f^	E	B
CO	2	Gas	2.72 × 10^−2^ b	0.11, [1.54]^e^,^g^	F	B
H_2_	2	Gas	0.16 × 10^−2^ b	3.00, [3.02]^d^	E	B
2‐Propanol	18	Liquid	Miscible	1.30, [4.34]^e^	C	C
2,3‐Butanediol	22	Liquid	Miscible	1.50, [6.14]^e^	C	C
Acetic acid	8	Liquid	Miscible	0.44, [3.25]^e^	C	C
Propionic acid	14	Liquid	Miscible	1.30, [6.79]^e^	C	C
Formic acid	2	Liquid	Miscible	0.55, [12.38]^e^	C	D
Oxalic acid	2	Solid	118	0.71, [35.21]^e^	C	D
Lactic acid	12	Liquid	Miscible	1.30, [9.61]^d^	D	D

^a^ Information gathered from the US National Institute of Standards and Technology (NIST, webbook.nist.gov/chemistry/).

^b^ Equilibrium concentrations for a partial pressure of 1 bar. Numbers calculated using the Henry coefficients from (Sander 2015).

^c^ Price between [] were calculated using the degree of reduction of the substances.

^d^ Prices gathered for products of renewable origin.

^e^ Prices gathered for products of nonrenewable origin.

^f^ Biogas price.

^g^ Captured from CO‐rich steel manufacturing off‐gas.

^h^ Sources are A: markets.businessinsider.com; B: icis.com; C: intratec.us; D: chemanalyst.com; E: iea.org; F: (Kildahl et al. 1974; 2023).

The potential of the substrates in Table 1 for industrial SCP production in the near future was assessed by classifying them into four categories, depending on their cost per electron and possible scalability issues. In Category A, glucose, glycerol, ethanol, and methanol were placed; their cost varies between 1.5 and 2.5 €/kmol_e‐_. The three gases, methane, CO, and H_2_, have similar costs as the substrates in Category A, but due to mass transfer limitations, the SCP production process may suffer from more difficult scalability and higher operation and fixed costs; thus, the gases were classified in Category B. 2‐Propanol, 2,3‐butanediol, acetic acid, and propionic acid were grouped in Category C since they are more expensive than substrates in Category A (3–7 €/kmol_e‐_) and also take part in smaller markets; although in the future, such markets could grow, which for now remains uncertain. Substrates with high prices (higher than 9 €/kmol_e‐_), such as formic, oxalic, and lactic acid, were grouped in Category D. The Category D substrates belong to even smaller markets, which are unlikely to grow to be competitive in the near future.

To identify technical factors that are important for the abovementioned process integration opportunities, a modeling approach was chosen. The aim of this paper is to develop a generic fermentation model that can be used to (i) evaluate the technical performance of the bioreactor when using alternative substrates, (ii) identify potential challenges to be faced during bioreactor scale‐up at an early stage, and (iii) to facilitate the choice of fermentation operation conditions. To develop the model, we chose ethanol as the example substrate for SCP production. Ethanol belongs to Category A in Table 1 and has already been explored at pilot and demonstration scales (Solomons and Litchfield 1983). Ethanol can be produced by electroreduction of CO_2_ (Wang et al. 2024), or H_2_ that is produced electrochemically can be used to convert CO_2_ to ethanol using chemical catalysis (Ding et al. 2020) or using fermentation (Lee et al. 2021).

In a later stage, when considering electrochemical production routes of the various alternative substrates from CO_2_, our model could facilitate a broader comparative feedstock analysis. This could include the technical feasibility, economic performance, and ecologic suitability of using the substrates for SCP production.

In this study, a bubble column operating at continuous mode was assumed to be suitable for aerobic SCP production at the industrial scale (Humbird et al. 2017; Jakobsen 1988).

For simplicity, SCP will be taken as equivalent to dry microbial biomass. The following key performance indicators were used here for SCP production (see the Notation for symbols):

- The dry biomass titer ($C_{x}$). High titers in continuous fermentations will save on the purchase costs of the bioreactor and the variable costs for the subsequent separation of cells from the fermentation broth.
- The biomass yield on ethanol = $\mu /(-q_{S}).$ High yields will save on costs related to ethanol use.
- The ethanol utilization in the bioreactor$\;=\left[1-\left(F_{L,\mathrm{out}}\cdot C_{S,\mathrm{out}}\right)/\left(F_{S,\mathrm{feed}}\cdot C_{S,\mathrm{in}}\right)\right]\cdot 100\%$. Although ethanol is valuable for other industrial processes that could be integrated with SCP production, the concentration in the liquid is too low for an energy‐efficient recovery, thus most of ethanol should be converted.
- The biomass production rate $R_{x}=D\cdot C_{x}\cdot M_{L}$ in mol_x_/h. Higher SCP production rates per unitary installed bioreactor capacity will reduce bioreactor fixed costs.
- The O_2_ utilization = $1-\left[\left(F_{G,\mathrm{out}}^{N}\cdot y_{O_{2},\mathrm{out}}\right)/\left(F_{G,\mathrm{in}}^{N}\cdot y_{O_{2},\mathrm{in}}\right)\right]\cdot 100\%$. Converting as much O_2_ as possible will save on costs related to O_2_ production and re‐compression.

The overall process performance will be limited by trade‐offs between the performance indicators and microbial and technical constraints. The following constraints will be addressed:

- The maximum specific growth rate $\mu^{\max}$ and maximum specific ethanol uptake rate $q_{S}^{\max}$ of the microbe used.

- The affinity of the microbe for ethanol and for O_2_.
- Potential inhibition of microbial growth by high CO_2_ concentrations.
- The achievable biomass concentration in the bioreactor.
- The achievable gas flow rate in the bioreactor.
- The achievable cooling rate in the bioreactor.
- The potential presence of concentration and temperature gradients in the bioreactor.

## 2 Methods

### 2.1 Bioreactor Configuration

**Figure 1:**
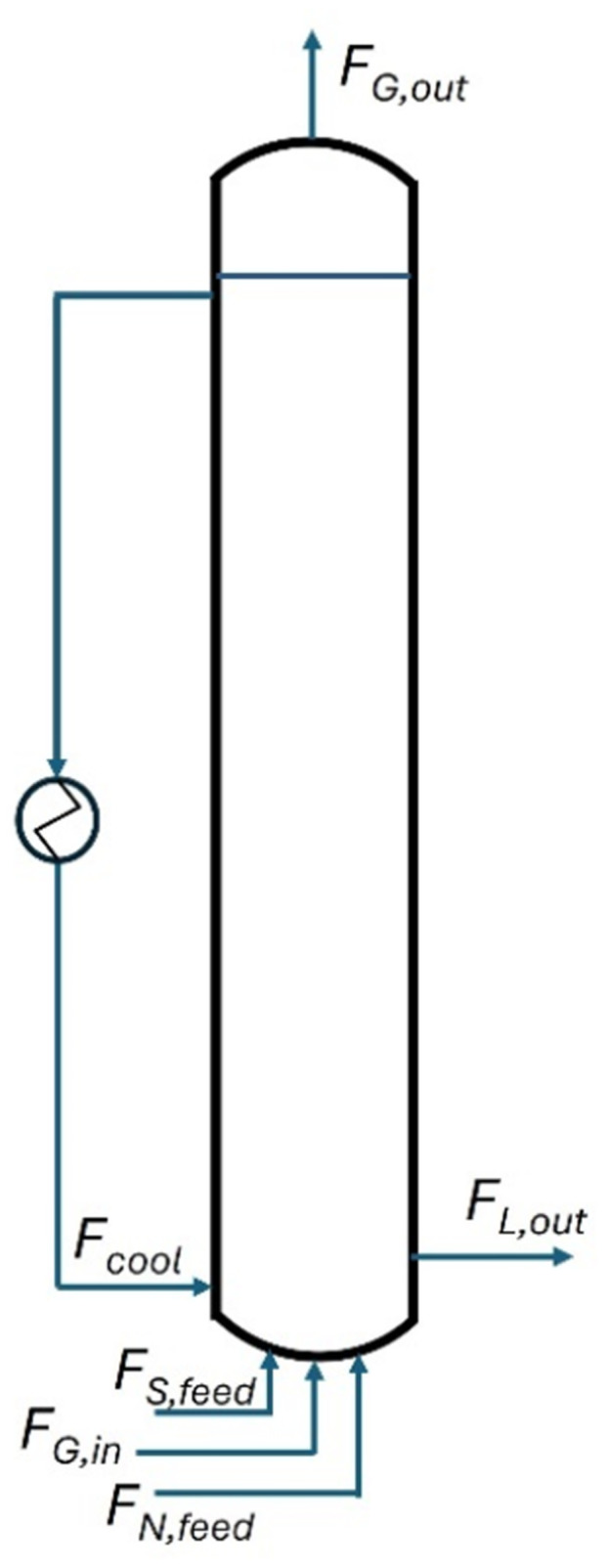
Schematic representation of the bubble column reactor used for SCP production. $F_{G,\mathrm{in}}$ and $F_{G,\mathrm{out}}$ refer to the gas in‐ and out‐flows; $F_{S,\mathrm{feed}}$ and $F_{N,\mathrm{feed}}$ refer to the inflow of ethanol (substrate S) and the nitrogen source (N), respectively, in water; $F_{L,\mathrm{out}}$ refers to the liquid outflow from the bioreactor; $F_{\mathrm{cool}}$ is the stream flowing through the external cooling loop.

Figure 1 shows a schematic representation of the bubble column bioreactor with the in‐ and out‐flow streams and the external cooling loop. In formulas, the flow symbol *F* will be given a superscript *M* for mass flows and *N* for mole flows.

The bioreactor volume ($V_{R}$) considered was 600 m^3^, which is in line with installed bubble columns for aerobic 1,3‐propanediol production (Bisgaard et al. 2022). This is smaller than sizes reportedly installed for aerobic SCP production in gas lift and U‐loop bioreactors, which are up to 1500 m^3^ (Goldberg 1985; Solomons and Litchfield 1983). The height‐over‐diameter ratio ($H_{R}/D_{R}$) was 6, a value commonly used for large bubble columns (Jakobsen 1988). The diameter ($D_{R}$) of 5.03 m for the cylindrical bioreactor vessel followed from its volume and the aspect ratio. Ethanol and ammonia solutions were fed as separate streams. Gases were supplied by sparging through orifice spargers. The fermentation broth, including cells, left the reactor as a single stream. Choices of the values of flow rates and input concentrations are discussed in Sections 2.2–2.7.

### 2.2 Model Assumptions

The following simplifying assumptions were used:

(i) The elemental composition for dry cells (x) is CH_1.8_O_0.5_N_0.2_ (Heijnen 2013), independent of growth conditions.
(ii) The cells and extracellular liquid are jointly regarded as the liquid phase (denoted as *L*).
(iii) The liquid and gas phases are assumed to be perfectly mixed along the bioreactor. Still, both assumptions are challenged and discussed in Section 3.7 using characteristic times.
(iv) The ungassed liquid density ($\rho_{L}$) is 1000 kg/m^3^.
(v) The affinity constant for O_2_ is typically 1–10 μmol/L, such that maintaining the dissolved O_2_ concentration above 0.069 mmol/L is assumed to suffice for keeping the growth rate zero order with respect to the dissolved O_2_ concentration (Villadsen et al. 2011). A dissolved O_2_ concentration ($C_{O_{2}}$) of 0.069 mmol/kg equals 30% of the saturation concentration of O_2_ at atmospheric conditions, which has been used experimentally in aerobic conversions (van Winden et al. 2022).
(vi) Ammonia serves as an N‐source. Growth is also negligibly reduced at dissolved ammonia concentrations above 1 g_NH3_/kg_L_.
(vii) The amounts of O_2_ and CO_2_ leaving the bioreactor as solutes in liquid are negligible relative to their amounts leaving with the off‐gas. Since the solubility of CO_2_ is 25 times higher than that of O_2_, this assumption is also challenged and discussed for CO_2_ in Section 3.3.
(viii) Evaporation of ethanol and NH_3_ is negligible due to their low residual concentrations in the bioreactor. Evaporation of water is considered.
(ix) For mass transfer calculations, the fermentation broth is assumed to be a coalescing liquid. This assumption may underestimate the overall mass transfer rate as the presence of ethanol will lead to a (locally) non‐coalescing broth near the sparger (Puiman et al. 2022). The consequences of potentially faster mass transfer rates due to inhibited coalescence are assessed in the sensitivity analysis (Section 3.8).

### 2.3 Operational Choices

(i) The bioreactor operates as a chemostat, a mode preferred for higher productivity and for maintaining constant growth conditions, thus constant SCP quality (Trinci 1994).
(ii) The fermentation temperature setpoint (T) is 30°C, a common temperature for yeast species growing on ethanol (see Table A1 in the Appendix A).
(iii) The overhead pressure ($p_{\mathrm{top}}$) is 1.2 bar (absolute). 0.2 bar is added to 1 bar of atmospheric pressure to prevent contamination of the broth by foreign microbes.
(iv) The aerated broth occupies 95% of the bioreactor volume to minimize the risk of foam from the bioreactor.
(v) NH_3_ is fed as an aqueous solution with a concentration of 200 g_NH3_/kg.
(vi) The pH is 6, a value common for yeast and which prevents high concentrations of carbonate.
(vii) Ethanol is fed pure or in solution with water.
(viii) The fraction of O_2_ in the gas inflow ($y_{O_{2},\mathrm{in}}$) is either 0.21 (air) or 1 (pure O_2_). The sparging of pure O_2_ is analyzed throughout most of the study, yet air sparging is used as a benchmark for brief comparisons.
(ix) The gas feed is dry.

### 2.4 Material Balances

The applied steady‐state material balances are given in Table 2, in terms of flow rates (F), transfer rates from gas to liquid ($N_{i}$), production rates ($R_{i}$), and mass of liquid ($M_{L}$).

**Table 2:** Steady‐state material balances in the SCP production bioreactor^a^.

Material	Phase		Equation	
O_2_	Gas	$0=F_{G,\mathrm{in}}^{N}y_{O_{2},\mathrm{in}}-F_{G,\mathrm{out}}^{N}y_{O_{2},\mathrm{out}}-N_{O_{2}}M_{L}$		Equation 1
CO_2_ b	Gas	$0=-F_{G,\mathrm{out}}^{N}y_{{\mathrm{CO}}_{2},\mathrm{out}}-N_{CO_{2}}M_{L}$		Equation 2
N_2_ c	Gas	$0=F_{G,\mathrm{in}}^{N}y_{N_{2},\mathrm{in}}-F_{G,\mathrm{out}}^{N}y_{N_{2},\mathrm{out}}$		Equation 3
Total	Gas	$0=F_{G,\mathrm{in}}^{N}-F_{G,\mathrm{out}}^{N}-{(N}_{O_{2}}+N_{{\mathrm{CO}}_{2}}+N_{W})M_{L}$		Equation 4
O_2_	Liquid	$0=N_{O_{2}}M_{L}+R_{O_{2}}$		Equation 5
CO_2_	Liquid	$0=N_{CO_{2}}M_{L}+R_{{\mathrm{CO}}_{2}}$		Equation 6
Ethanol	Liquid	$0=F_{S,\mathrm{feed}}^{M}C_{S,\mathrm{in}}-F_{L,\mathrm{out}}^{M}C_{S}+R_{S}$		Equation 7
Biomass	Liquid	$0=R_{x}-F_{L,\mathrm{out}}^{M}C_{x}$		Equation 8
NH_3_	Liquid	$0=F_{N,\mathrm{feed}}^{M}C_{{\mathrm{NH}}_{3},\mathrm{in}}-F_{L,\mathrm{out}}^{M}C_{{\mathrm{NH}}_{3}}+R_{{\mathrm{NH}}_{3}}$		Equation 9
Total	Liquid	$0=F_{S,\mathrm{feed}}^{M}+F_{N,\mathrm{feed}}^{M}-F_{L,\mathrm{out}}^{M}+{(N}_{O_{2}}+N_{{\mathrm{CO}}_{2}}+N_{W})M_{L}$		Equation 10

^a^ Water balance is not included because it is not used for solving the material balances; the total material balances serve this purpose.

^b^ CO_2_ present in the air is not included in the balance.

^c^ N_2_ balance is only used when air is used as an O_2_ source.

### 2.5 Transfer Rates

The mass‐specific O_2_ transfer rate ($N_{O_{2}}$) depends on both the mass transfer coefficient (${\left[k_{L}a\right]}_{O_{2}}$) and the driving force (i.e., the difference between the logarithmic mean equilibrium concentration at the bubble surface [$C_{O_{2}}^{*}$] and the actual concentration of O_2_ in the bulk of the liquid [$C_{O_{2}}$]):

(11) $$N_{O_{2}}={\left[k_{L}a\right]}_{O_{2}}\left(C_{O_{2}}^{*}-C_{O_{2}}\right)$$

The equilibrium concentration depends on the local O_2_ partial pressure in the gas phase and its Henry coefficient ($K_{H}$) in pure water. Since the gas phase is assumed to be ideally mixed, the gas in the bioreactor will have the same composition as the off‐gas.

(12) $$C_{O_{2}}^{*}=K_{H,O_{2}}y_{O_{2},\mathrm{out}}p$$

The mean superficial gas velocity ($v_{\mathrm{sG}}^{\mathrm{mean}}$, in m/s) determines the ${\left[k_{L}a\right]}_{O_{2}}$ (in s^−1^), assuming the broth is a coalescing liquid. Similarly, the $v_{\mathrm{sG}}^{\mathrm{mean}}$ also determines the gas hold‐up ($\varepsilon_{G}$) (Heijnen and Van't Riet 1984; Van't Riet and Tramper 1991):

(13) $${\left[k_{L}a\right]}_{O_{2}}=1.022^{\left(T-20\right)}\left(0.32{v_{\mathrm{sG}}^{\mathrm{mean}}}^{0.7}\right)$$

(14) $$\varepsilon_{G}=0.6{v_{\mathrm{sG}}^{\mathrm{mean}}}^{0.7}$$

The mean superficial gas velocity is the logarithmic mean between the velocities at the top and at the bottom of the column. These values are related to the respective molar gas flow rate $F_{G}^{N},$ at the top and bottom, according to Van't Riet and Tramper (1991):

(15) $$v_{\mathrm{sG}}=\frac{F_{G}^{N}\left(\frac{\mathrm{RT}}{p}\right)}{\frac{\pi }{4}{D_{R}}^{2}}$$

(16) $$v_{\mathrm{sG}}^{\mathrm{mean}}=\frac{v_{\mathrm{sG}}^{\mathrm{top}}-v_{\mathrm{sG}}^{\mathrm{bot}}}{\ln\left(\frac{v_{\mathrm{sG}}^{\mathrm{top}}}{v_{\mathrm{sG}}^{\mathrm{bot}}}\right)}$$

The hydrostatic pressure at the bottom of the column was calculated by summing the top pressure and the hydrostatic pressure of the liquid column; the latter follows from the height of aerated liquid using $\varepsilon_{G}$ and the assumption that 95% of the bioreactor volume was filled by the gas–liquid mixture.

The CO_2_ mass transfer rate was calculated similarly to the O_2_ transfer rate, but using a mass transfer coefficient corrected for CO_2_ (Heijnen and Van't Riet 1984):

(17) $${\left[k_{L}a\right]}_{CO_{2}}={\left[k_{L}a\right]}_{O_{2}}{\left(\frac{D_{CO_{2}}}{D_{O_{2}}}\right)}^{1/2}$$

Water was assumed to be at equilibrium between the gas and liquid phases; hence the rate of transfer by evaporation (${-N}_{W}$) depends on its saturated vapor pressure $p_{W}^{\mathrm{sat}}$ and the off‐gas flow rate:

(18) $$-N_{W}=\frac{p_{W}^{\mathrm{sat}}}{p}\frac{F_{G,\mathrm{out}}^{N}}{M_{L}}=y_{W,\mathrm{out}}\frac{F_{G,\mathrm{out}}^{N}}{M_{L}}$$

The used parameter values are given in Table A3, in the Appendix A.

### 2.6 Production Rates

Mass‐specific production rates ($r_{i}$, in (mol_i_/(kg_L_ h)) were calculated by multiplying biomass‐specific production rates ($q_{i}$, in (mol_i_/(h mol_x_))) by biomass concentration ($C_{x}$, in (mol_x_/kg_L_)). Production rates ($R_{i}$, in (mol_i_/h)), in the bioreactor, were derived by further multiplication with the liquid mass:

(19) $$R_{i}=q_{i}C_{x}M_{L}$$

The specific uptake rate of the substrate ($q_{S}$) depends hyperbolically on substrate concentration ($C_{S})$:

(20) $$q_{S}=q_{S}^{\max}\left(\frac{C_{S}}{K_{S}+C_{S}}\right)$$

Adopting the Herbert–Pirt equation (Pirt 1965), the consumed substrate is used for growth and maintenance (rates *µ* and $m_{S}$, respectively):

(21) $${-q}_{S}=\frac{1}{Y_{x/S}^{\max}}\mu +m_{S}$$

The values used for the parameters $q_{S}^{\max}$, $K_{S}$, $Y_{x/S}^{\max}$, and $m_{S}$ can be found in Section A.1 of the Appendix A, in addition to the procedures followed to define them. The procedure followed to define the stoichiometry of the process reaction is also described in Section A.1 of the Appendix A.

### 2.7 Solving the Material Balances

The MATLAB code used (see Data Availability Statement) shows details of the solution method. The first step for solving the material balances was to define the process reaction stoichiometry as a function of the growth rate. A growth rate (μ) was selected that is favorable for producing cells at a large scale, such that the biomass yield approaches the maximum while the ethanol concentration is low, to avoid wasting ethanol in the liquid outflow stream. The selected μ (equaling the dilution rate for the continuous fermentation) fixes the ratio between CO_2_ production and O_2_ consumption ($R_{CO_{2}}/R_{O_{2}}$), independently of the gas flow rate. The procedure for finding the process reaction stoichiometry as a function of μ is described in Section A.1 of the Appendix A.

Using the ratio $R_{CO_{2}}/R_{O_{2}}$, the gas‐phase material balances were solved iteratively per value of $v_{\mathrm{sG}}^{\mathrm{top}}$ by using the equations discussed in Section 2.5. The iterations used the comparison between O_2_ transfer rate ($N_{O_{2}}$) calculated from two different equations (i.e., from the total material balance in the gas phase [Equation 4] and from the product between $k_{L}a$ and the driving force for mass transfer [Equation 11]) as the objective function (i.e., the difference between the two calculations should be zero). The decision variable for the iterations was $v_{\mathrm{sG}}^{\mathrm{bot}}$.

For a favorable value of $N_{O_{2}}$, the rates of the other reaction components were calculated using the reaction stoichiometry. The ungassed liquid mass was then obtained through the gas hold‐up. The gas hold‐up was derived from the superficial gas velocity, which was calculated from the gas in‐ and out‐flow rates. The liquid outflow rate came from dividing the liquid mass by the dilution rate; the biomass concentration was obtained by dividing the biomass production rate by the liquid outflow rate. The biomass production rate and the stoichiometry were used to calculate the required inflow rates of the substrate and ammonia feeds, as well as the substrate feed concentration.

The values of all the input variables are mentioned in Sections 2.1, 2.2, and 2.3.

### 2.8 Heat Balance and Heat Transfer

The steady‐state heat balance includes terms for the heat of reaction ($Q_{r}$), for evaporation of water ($Q_{\mathrm{evap}}$), and for heat removed by cooling ($Q_{\mathrm{cool}}$):

(22) $$0=Q_{r}-Q_{\mathrm{evap}}-Q_{\mathrm{cool}}$$

The feed streams were assumed to be at fermentation temperature. In addition, the sparging of compressed gas contributes only negligible amounts of energy to the heat balance in a bubble column fermentation process (Roels and Heijnen 1980).

The heat of the reaction was calculated from the O_2_ consumption. As proposed in Roels (1983), 460 kJ of heat are generated per mole of O_2_ consumed:

(23) $$Q_{r}=460\cdot (-R_{O_{2}})$$

The energy withdrawn by the evaporation of water depends on both the amount evaporated and the heat of evaporation of water (${∆}_{\mathrm{evap}}H$):

(24) $$Q_{\mathrm{evap}}=F_{G,\mathrm{out}}y_{W,\mathrm{out}}{∆}_{\mathrm{evap}}H$$

Equations 22 to 24 allowed the calculation of the heat to be removed by cooling. The need for an external cooling system was identified by calculating the cooling area that can be provided by an internal coil, which is 474 m^2^. That area was calculated assuming (i) a coil pipe diameter equal to $D_{R}$/30 (Towler and Sinnott 2013), (ii) the pitch equal to twice the pipe diameter (Towler and Sinnott 2013), and (iii) the number of turns equal to the height of aerated liquid divided by the sum of the pipe diameter and the pitch. In the present study case, such an area was found to be between 1.1 and 3.5 m^2^/m^3^ for the range of superficial gas velocities. The external cooling loop then consists of shell‐and‐tube (SAT) heat exchangers, with the fermentation broth and chilled water in countercurrent flow. The design of the largest SAT heat exchanger (Walas 1990) was taken to identify potential sources of stress to be faced by cells when flowing through the cooling loop. Such a design has a maximum tube length of 6.1 m, for stainless steel tubes with an internal diameter of 19 mm; the tubes are organized in a triangular arrangement. This resulted in 1269 tubes within one heat exchanger of ca. 430 m^2^ of area available for heat transfer.

The heat transfer coefficient (U) suited for this system was 1.4 kW/(m^2^K) (Towler and Sinnott 2013). The broth was set to cool from 30°C to 15°C, and the chilled water was set to heat up from 5°C to 20°C, leading to a mean gradient ($∆T_{\mathrm{lm}}$) of 15°C. The required cooling area $A_{\mathrm{cool}}$ followed from:

(25) $$Q_{\mathrm{cool}}={U\;A}_{\mathrm{cool}}\;{∆T}_{\mathrm{lm}}$$

The rate at which the liquid flows through the cooling loop $F_{L,\mathrm{cool}}$ was calculated using the heat capacity of water *c*_*p*_ and the temperature drop (${∆T}_{L,\mathrm{cool}}$) of 15°C:

(26) $$Q_{\mathrm{cool}}=F_{L,\mathrm{cool}}\;c_{p}{\;∆T}_{L,\mathrm{cool}}$$

The velocity in the tubes was calculated from the flow rate, using the tubes' cross‐sectional internal area and the number of tubes per heat exchanger.

### 2.9 Characteristic Times

Characteristic times were calculated to better understand the interactions between hydrodynamics and other mechanisms in the bioreactor. Learning of such interactions in this early‐stage assessment is regarded useful for identifying potential challenges during bioreactor scale‐up. In addition, the characteristic times were also used to verify the assumption of perfectly mixed gas and liquid phases. The mechanisms, descriptions, and equations that were used are given in Table A4.

## 3 Results and Discussion

### 3.1 Choice of Dilution Rate

**Figure 2:**
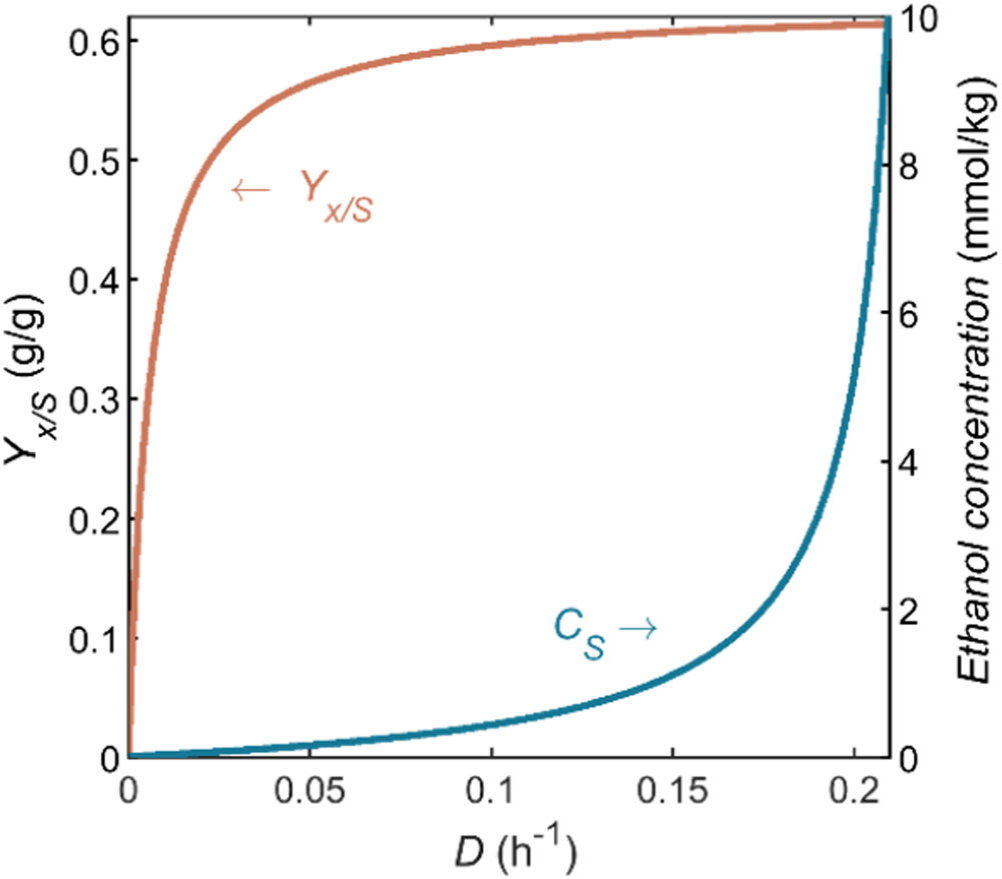
Yield of biomass on ethanol ($Y_{x/S}$) and residual ethanol concentration ($C_{S}$) as a function of the dilution rate (D) during ethanol‐limited growth on ethanol in a chemostat.

The stoichiometry of the reaction depends on the growth rate (μ), which equals the dilution rate (D) through the biomass balance. Figure 2 shows the biomass yield on ethanol ($Y_{x/S}$) as a function of the dilution rate. The difference between this yield and the maximum yield (0.63 g_x_/g_S_) is due to ethanol use for maintenance. The fraction of ethanol that is directed toward maintenance is minimized at high dilution rates. To achieve high yields of biomass on ethanol, the dilution rate range to be used for SCP production is 0.07–0.2 h^−1^. For growth on glucose, values from 0.1 to 0.2 h^−1^ have been reported (Solomons and Litchfield 1983; Trinci 1994; Whittaker et al. 2020). At even higher dilution rates, the maximum growth rate ($\mu^{\max}$ is 0.22 h^−1^) is approached. Close to this maximum, biomass washout may already occur due to unintended fluctuations in operation conditions.

A related issue is substrate utilization. In a carbon‐limited chemostat, the residual substrate concentration ($C_{S}$) is generally low; thus, almost no substrate is unused, which is favorable for the overall process performance. An exception is the situation when D is only slightly below $\mu^{\max}$, another reason for avoiding dilution rates in the higher range. Therefore, a D of 0.15 h^−1^ is selected, and it will be used in the next sections of this study. Hence, the stoichiometry resulting from those conditions applies, as shown in Table 3. The ethanol concentration in the bioreactor is thus 1.1 mmol/kg_L_ (0.05 g/kg_L_). Literature suggests no growth inhibition by ethanol for concentrations up to 10 and 4 g/kg_L_ (217 and 87 mmol/kg_L_) (Preez et al. 1981; Paalme et al. 1997), respectively; thus, 0.05 g/kg_L_ ethanol will not constrain the reaction rate.

**Table 3:** Stoichiometric coefficients of the process reaction for growth on ethanol at a specific growth rate of 0.15 h^−1^.

Reaction component	Coefficient [mol_i_/mol_x_]
Biomass	1.00
Ethanol	−0.88
O_2_	−1.59
CO_2_	0.76
H_2_O	2.04
NH_3_	−0.20

Since the ethanol consumed for cell maintenance is low at D 0.15 h^−1^ (about 4% ethanol), the model is largely insensitive to the value of the maintenance coefficient used in the calculations (0.005 mol_S_/[mol_x_ h]).

### 3.2 O_2_ Transfer and Utilization

**Figure 3:**
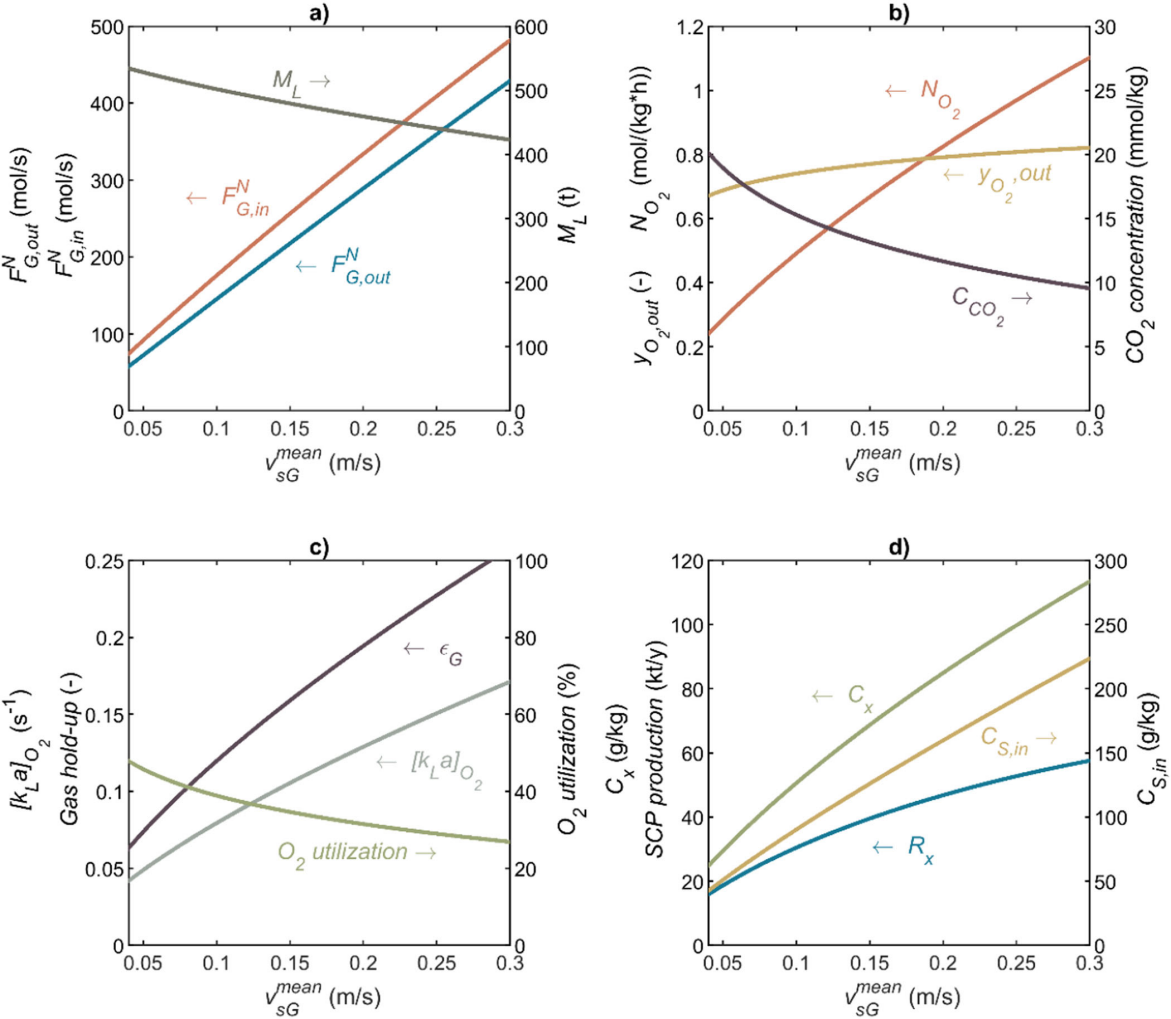
Main operation features of the SCP production bubble column bioreactor when pure O_2_ is sparged and the dilution rate is 0.15 h^−1^. Features are (a) gas flow entering and leaving the bubble column ($F_{G,\mathrm{in}}$ and $F_{G,\mathrm{out}}$) and liquid mass ($M_{L}$); (b) O_2_ transfer rate ($N_{O_{2}}$), O_2_ molar fraction in off‐gas ($y_{O_{2},\mathrm{out}}$) and CO_2_ concentration in liquid ($C_{{\mathrm{CO}}_{2}}$); (c) gas hold‐up ($\varepsilon_{G}$), O_2_ transfer coefficient (${\left[k_{L}a\right]}_{O_{2}}$), and O_2_ utilization; (d) cells concentration ($C_{x}$), cells productivity ($R_{x}$), and ethanol concentration in the feed stream ($C_{S,\mathrm{in}}$).

Productivities of aerobic fermentations, operating at high dilution rates, are constrained by the maximum achievable O_2_ transfer rate (Noorman et al. 2018). In a bubble column, high mass transfer rates are obtained when bubbles flow in a turbulent flow pattern: the heterogeneous flow regime. Such regime is generated in industrial‐size columns when superficial gas velocities ($v_{\mathrm{sG}}^{\mathrm{mean}}$) are above 0.04–0.08 m/s up to the flooding of the column (when the gas blows out the liquid), at $v_{\mathrm{sG}}^{\mathrm{mean}}$ on the order of 1 m/s (Van't Riet and Tramper 1991). Thus, we considered $v_{\mathrm{sG}}^{\mathrm{mean}}$ to be in the range between 0.04 and 0.30 m/s. The latter value is significantly lower than the flooding conditions and limits the applicability of the model used for calculating $k_{L}a$ (Equation 13) (Heijnen and Van't Riet 1984). As shown in Figure 3a, which applies to sparging pure O_2_, the gas outflow is smaller than the gas inflow because more O_2_ is consumed than CO_2_ is produced, as the respiratory quotient ($q_{CO_{2}}/-q_{O_{2}}$) of the process is 0.48 (see Table 3). Within the used range for $v_{\mathrm{sG}}^{\mathrm{mean}}$, the gas hold‐up increases from 0.06 to 0.26 (Figure 3c), resulting in a decrease of 21% in liquid mass in the column (see $M_{L}$ in Figure 3a). Large changes are also seen in the mass transfer coefficients and overall O_2_ transfer rates (Figure 3c,b, respectively). Calculated ${\left[k_{L}a\right]}_{O_{2}}$ and $N_{O_{2}}$ range between 150 and 617 h^−1^ and between 0.24 and 1.09 mol/(kg_L_ h), respectively. The latter $N_{O_{2}}$ value is well beyond typical values for fermentations that use air; yet, it is deemed possible (Van't Riet and Tramper 1991). Therefore, the values shown in Figure 3 might be achievable due to the enhancement of the driving force for mass transfer caused by pure O_2_.

The large O_2_ gas flows needed for promoting mass transfer also lead to poor utilization of the O_2_ supplied (ranging from 48% to 27% for $v_{\mathrm{sG}}^{\mathrm{mean}}$ increasing from 0.04 to 0.30 m/s [Figure 3c]). The reason for poorer utilization upon increasing $v_{\mathrm{sG}}^{\mathrm{mean}}$ is that *k*_L_ *a* does not increase proportionally with $v_{\mathrm{sG}}^{\mathrm{mean}}$ (see Equation 13), such that the amount of O_2_ sparged into the bioreactor increases faster than the amount of O_2_ transferred, which leads to a larger O_2_ loss. Releasing the off‐gas to the atmosphere would be a waste because of two reasons: (i) the off‐gas contains more O_2_ than that present in the air ($y_{O_{2},\mathrm{out}}$ ranges between 0.67 and 0.82, see Figure 3b) and (ii) the remaining fraction is mostly CO_2_ (there is also a small fraction of water). The O_2_ remaining after CO_2_ capture can be recycled to the fermentation to reduce the need for fresh O_2_, whereas the captured CO_2_ can be sent to the upstream ethanol production process. Recovering O_2_ from fermentation off‐gas and recycling this O_2_ to fermentation have been proposed for aerobic fermentation of *Ralstonia eutropha* and was shown to be favorable (Chang et al. 2010).

Complete O_2_ utilization is not possible since minimum dissolved O_2_ concentrations must be maintained in the liquid to prevent O_2_ limitation of the microbes, and the driving force for gas‐to‐liquid O_2_ transfer should be kept.

If air were used instead of pure O_2_, the O_2_ utilization would be slightly lower than that when pure O_2_ is used due to the overall slower O_2_ transfer. For the model input values of Table A5, this can be seen in Table A6. Furthermore, using air introduces N_2_ into the system, which might accumulate in the system if it is not completely separated from O_2_ after the CO_2_‐capturing process.

Despite the possibility to recycle unused O_2_ when using pure O_2_, a higher O_2_ utilization is still desired because of the cost savings associated with its recycling. Improving the gas injection systems (as in Groen et al. 2005) and using a taller bioreactor are options that could be further assessed.

### 3.3 CO_2_ Toxicity

The dissolved CO_2_ concentration ($C_{{\mathrm{CO}}_{2}}$) is expected to range from 20 to 9.6 mmol/kg_L_ for $v_{\mathrm{sG}}^{\mathrm{mean}}$, increasing from 0.04 to 0.30 m/s (Figure 3b). It is uncertain if such CO_2_ concentrations might inhibit ethanol‐based fermentation. In general, available data on growth inhibition by CO_2_ are too scattered to assume an inhibition model equation (Blombach and Takors 2015; Jones and Greenfield 1982). The data range from smooth anaerobic growth of *Saccharomyces cerevisiae* on carbohydrates with 100% CO_2_ in off‐gas, leading to $C_{{\mathrm{CO}}_{2}}$ of 29 mmol/kg_L_ at 1 bar (Della‐Bianca et al. 2013), to using CO_2_ in food production to inhibit the growth of unwanted fungi and bacteria (Dixon and Kell 1989). However, the aerobic growth of *S. cerevisiae* on a carbohydrate could be considered as a reference to provide perspective on the effect of $C_{{\mathrm{CO}}_{2}}$ on microbial growth. *S. cerevisiae* became sensitive to $C_{CO_{2}}$ at values above 13 mmol/kg_L_ (Chen and Gutmanis 1976). For pure O_2_, using $v_{\mathrm{sG}}^{\mathrm{mean}}$ in excess of 0.14 m/s prevents $C_{{\mathrm{CO}}_{2}}$ from exceeding 13 mmol/kg, according to Figure 3b. The next sections will show that such high values of $v_{\mathrm{sG}}^{\mathrm{mean}}$ will be favorable, such that we decided that it was not essential to include inhibition by CO_2_ in the model. Still, the actual selection of a microbial strain for SCP production and the design of a bioreactor should consider the effect of $C_{CO_{2}}$ on microbial activity, yields, and viability. Even though not reported for high CO_2_ concentrations, the adaptiveness of microbes to adverse conditions might be exploited in adaptive lab evolution approaches (Hirasawa and Maeda 2023). Air could be mixed with pure O_2_ to limit O_2_ transfer and keep $C_{CO_{2}}$ below the eventual critical value. The identified knowledge gap on CO_2_ toxicity calls for new experimental studies on this topic, under conditions that are favorable for SCP production.

Lastly, the stepwise procedure (see Section 2.7) adopted for solving the mass balances required an assumption relevant to the calculated $C_{CO_{2}}$: the CO_2_ production rate ($R_{CO_{2}}$) equals CO_2_ transfer rate ($N_{CO_{2}}$). That assumption neglects the CO_2_ that leaves the bioreactor as a solute in the liquid phase, inducing an error in the carbon balance that ranges between 7.0% and 0.1% when $v_{\mathrm{sG}}^{\mathrm{mean}}$ goes from 0.04 to 0.30 m/s. In other words, more carbon leaves the reactor than that supplied in the inflow streams. Thus, it is advised that all mass balance equations are solved at once in future implementations of the model presented here.

### 3.4 Biomass Concentration and Production Rate

At high dilution rates (including the chosen 0.15 h^−1^), the rate at which ethanol can be supplied without accumulating, and thereby the rate at which cells are produced, is determined by the rate at which O_2_ is supplied. Considering the process reaction stoichiometry (see Table 3), 1/1.59 moles of cells are produced by each mole of O_2_ transferred from the gas to the liquid. The concentration of cells ($C_{x}$) at a defined production rate ($r_{x}$) is determined by D. Therefore, $C_{x}$ is proportional to $N_{O_{2}}$, where the proportionality is determined by the yield of cells on O_2_ divided by D. For example, at $v_{\mathrm{sG}}^{\mathrm{mean}}$ of 0.30 m/s, $N_{O_{2}}$ is 1.09 mol/(kg_L_ h); then, cells are produced at a rate of 0.69 mol/(kg_L_ h), which dividing by D, leads to a maximum $C_{x}$ of 114 g_x_/kg_L_ (see Figure 3d). For comparison, Vieira‐Lara et al. (2024), using *Candida jadinii*, show that at least 100 g_x_/kg_L_ can be achieved using ethanol‐limited fed‐batch fermentation. Our maximum $C_{x}$ corresponds to a biomass production rate of 7198 kg/h (58 kt/y on a 330 days‐per‐year basis). For comparison, using enhanced O_2_ transfer, a growth rate of 16 g_x_/(kg_L_ h) of *S. cerevisiae* on glucose has been reported by Groen et al. (2005), which is only slightly lower than what is feasible with ethanol according to our model (17 g_x_/[kg_L_ h]).

The found maximum $C_{x}$ conveniently falls below another physical constraint for $C_{x}$ that is expected at 150 g_x_/kg_L_. Above such concentrations, O_2_ transfer may be hampered due to increases in the viscosity of the liquid phase (Van't Riet and Tramper 1991). In addition, at a $C_{x}$ of 150 g_x_/kg_L_, the wet cell mass concentration is about 500 g/kg_L_ since cells are composed of about 70% water (Feijó Delgado et al. 2013). This 500 g/kg_L_ is not far from the maximum packing of spheres in a volume and leaves barely enough water in the bioreactor for mass transfer (Fu et al. 2014; Fuchs et al. 2002).

### 3.5 Choice of the Ethanol Feed Concentration

To prevent dissolved O_2_ rather than ethanol kinetically limiting growth, the amount of supplied ethanol should be such that the O_2_ consumption required to metabolize that feed rate does not exceed the O_2_ transfer rate at the set dissolved O_2_ level. The concentration of ethanol in the feed should ensure that a $C_{x}$ of 114 g/kg is achieved while keeping the average $C_{S}$ at 0.05 g/kg in the bulk of the liquid phase.

For D and $C_{x}$ at values of 0.15 h^−1^ and 114 g/kg, respectively, the calculation sequence of the ethanol concentration in the feed proceeded as follows: the dilution rate determines that the liquid outflow rate is 63 t/h. The reaction stoichiometry fixes the amounts of converted O_2_ and produced CO_2_; hence, the net flow from the gas phase to the liquid is obtained, which is substantial (5 t/h). The reaction stoichiometry also fixes the amount of NH_3_ that will be converted, and the mass flow rate of the nitrogen source feed is found (5 t/h). The ethanol‐containing feed flow (53 kg/h) closes the gap between inflows and outflows. The ethanol inflow should support an ethanol consumption rate ($-r_{S}$) of 0.605 mol_S_/(kg h), leading to the need for a $C_{S,\mathrm{in}}$ of 224 g/kg.

When using an ethanol concentration in the feed of only 224 g/kg, only limited water removal would be needed after the upstream ethanol production process. The integration of an ethanol production process based on electrolysis and CO_2_ capture, where ethanol may be produced at concentrations around 50 g/kg (Phillips et al. 1993), with the subsequent SCP production shows one potential advantage compared to a SCP production process using pure ethanol as feedstock since it is well‐documented that water removal from ethanol requires substantial energy (Janković et al. 2023).

Lastly, after the ethanol inflow and outflow rates for the bubble column are known, the ethanol utilization can be found, which turns out to be more than 99.9%.

### 3.6 The External Cooling Loop

The aerobic fermentation will produce a large amount of heat, and without sufficient cooling capacity the temperature would increase to values outside its optimum range (Noorman et al. 2018). The heat load (Q) is proportional to the O_2_ consumption rate. Q is then also proportional to the biomass production rate, which should be as high as possible. Therefore, we used the highest achievable rate of heat production, which occurs at $v_{\mathrm{sG}}^{\mathrm{mean}}$ of 0.30 m/s and a $-r_{O_{2}}$ of 1.09 mol/(kg_L_ h), for the design and assessment of the cooling system. The required cooling duty is then 59 MJ, which, considering the operation temperature of 30°C, should be provided by chilled water in a heat exchanger external to the bioreactor. Using the cooling system assumptions mentioned in Section 2.8, the required cooling area is 2805 m^2^. Thus, seven SAT heat exchangers of 400 m^2^ (which is below the maximum value of ca. 430 m^2^ found in Walas [1990]), connected in parallel, are needed. The total flow rate of the fermentation broth through the cooling loop is 939 kg/s. That is 53 times the liquid outflow rate and 8 passes per hour (or 1 pass every 7.5 min). For comparison, 4 passes per hour were reported by Groen et al. (2005) for cooling industrial‐scale aerobic growth of *S. cerevisiae* on glucose. Lastly, the flow rate through the cooling loop was found to be 18 times lower than the internal flow rate providing liquid mixing, the latter being calculated with Equation 27 (Heijnen and Van't Riet 1984). Thus, $F_{\mathrm{cool}}$ provides only marginal additional input to mixing the reactor contents.

(27) $$F_{\mathrm{mix},L}^{V}=0.3{D_{R}}^{\frac{5}{3}}{F_{G,\mathrm{mean}}^{V}}^{\frac{1}{3}}g^{\frac{1}{3}}$$

In addition to the 15°C drop in temperature every 7.5 min, which is by itself remarkably severe for microorganisms, two other potential sources of stress for microbes are assessed. One is a comparison between the potential residence time within the cooling loop and the time cells take to deplete both the substrate and the O_2_. The second one is the shear stress due to biomass flowing through the loop piping and the pump.

Addressing the first possible source of stress, assuming zero‐order kinetics, cells may take up all the dissolved O_2_ and the substrate in a time frame from 1 to 0.2 s and from 30 to 6.6 s, respectively, when $v_{\mathrm{sG}}^{\mathrm{mean}}$ increases from 0.04 to 0.30 m/s. Then, assuming the liquid will take one pass within the heat exchanger tubes, the flow velocity is 0.41 m/s when $v_{\mathrm{sG}}^{\mathrm{mean}}$ is highest. It will take about 39 s for the liquid to flow through the 6 m of heat exchanger length plus an assumed extra 10 m of piping connecting the bioreactor with the heat exchanger. Cells will then surely face O_2_ depletion, and since there will be no substrate consumption without O_2_, the substrate will not be depleted. The flow velocity may be increased by increasing the number of passes within the tubes. If 4 passes are used, the flow velocity will be around 1.6 m/s, and the residence time in the loop will lower to 20 s, taking (4.6 + 10) meter‐path. Thus, the effects of short‐term O_2_ depletion on cells should be assessed experimentally.

Now assuming a viscosity of 10 mPa s for the liquid phase, the flow velocities of 0.4 and 1.6 m/s used in the analysis above will provide a shear stress of 0.21 and 0.85 Pa, respectively. Considering that the viability of *S. cerevisiae* and *Escherichia coli* cells was reported to be affected at shear stress above 1300 Pa (Lange et al. 2001), the flow of liquid through the tubes is not a source of stress for cells. It is also unlikely that the shear stress provided by the piping fittings will be significant if designed properly.

Lastly, the massive flow rate through the loop also requires a large pump between the bioreactor and the heat exchangers. The pipe connecting the loop with the bioreactor may have a diameter of 2.3 or 0.6 m for the two flow velocities used above (0.4 and 1.6 m/s). Then, assuming that the axial flow pump impeller has a diameter of 0.6 m and that it leaves a clearance of 0.3 mm between the impeller blades tips and the pump casing (as used by Shen et al. [2019]), the shear stress faced by cells passing through the pump may fall between 63 and 628 Pa, for the pump impeller rotating between 60 and 600 rpm, respectively. The periodic circulation through such a high shear zone may become another relevant source of stress for microbes, as also acknowledged by others (Chisti 1999; Van't Riet and Tramper 1991).

### 3.7 Potential Existence of Concentration Gradients

**Figure 4:**
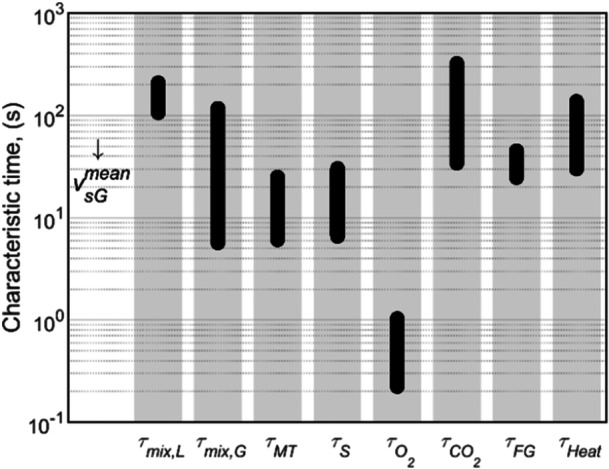
Characteristic times calculated at a specific growth rate of 0.15 h^−1^ and for the mean superficial gas velocity ranging from 0.04 to 0.30 m/s. The values of all the characteristics' times decrease as the superficial gas velocity increases.

The model was built assuming that the liquid and gas phases would be perfectly mixed. However, it is known that large‐scale bioreactors have limited mixing (Lara et al. 2006; Nadal‐Rey et al. 2021; Noorman 2015); thus, concentration and temperature gradients are likely present (Noorman et al. 2018). The potential for finding gradients was assessed by comparing the characteristic times of mixing with those related to the conversion process and mass transfer. Since the comparison is based on largely simplified calculations, the potential for finding gradients is only flagged when the characteristic times differ by more than one order of magnitude (Groen et al. 2005; Noorman 2015). Figure 4 shows the characteristic times calculated at $v_{\mathrm{sG}}^{\mathrm{mean}}$ values ranging from 0.04 to 0.30 m/s.

In general terms, the characteristic time for liquid mixing ($\tau_{\mathrm{mix},L}$) is one order of magnitude larger than that of substrate consumption ($\tau_{S}$) (see Figure 4). Therefore, substrate concentration gradients can be expected between the feed point (if located at the bottom of the bioreactor like in Figure 1) and the top of the liquid column. Implementing extra substrate feed points along the height of the reactor column might be a good solution to reduce the size of $C_{S}$ gradients along the height of the bioreactor. Extra feeding points may also help to reduce the effects of potential inhibition caused by substrate feeding at 224 g/kg because the smaller substrate feed flow rates will mix faster with the bulk of the liquid.

Although the $\tau_{\mathrm{mix},L}/\tau_{O_{2}}$ ratio is in the range of 100, the existence of large gradients in $C_{O_{2}}$ areis not expected. Opposite to substrate feeding, which may occur through a limited number of points, O_2_ transfer from the gas bubbles to the liquid occurs throughout the whole mass of liquid. Yet, small gradients can be found for the O_2_ transfer rates since the $k_{L}a$ and the O_2_ equilibrium concentration change with the height of the liquid column. Overall, the $N_{O_{2}}$ value at the bottom of the bioreactor can be around 3 times higher than that at the top, if the gas phase is perfectly mixed. A comparison between the characteristic times for the gas phase mixing ($\tau_{\mathrm{mix},G}$) and that of its flow across the column ($\tau_{F_{G}}$) suggests that mixing occurs only 4 times faster than gas flow. Thus, the gas composition may be between the conditions of a perfectly mixed system and a plug‐flow gradient; thus, the ratio between $N_{O_{2},\mathrm{bot}}$ and $N_{O_{2},\mathrm{top}}$ will be larger than 3. The implementation of improvements in O_2_ consumption will make $N_{O_{2}}$ gradients even larger. For instance, if $y_{O_{2},\mathrm{out}}$ was 0.1 while still sparging pure O_2_, the ratio between $N_{O_{2},\mathrm{bot}}$ and $N_{O_{2},\mathrm{top}}$ will rise to 4 for a perfectly mixed gas phase and to 56 for a gas phase behaving as plug flow. The true behavior will lie in between those two values.

Moreover, the characteristic times for CO_2_ and heat production are in line with or larger than $\tau_{\mathrm{mix},L}$ (see Figure 4), indicating that significantly smaller gradients may be expected for the broth temperature and CO_2_ concentration compared to those of $C_{S}$ and $N_{O_{2}}$.

Summarizing, three environments may be expected within a large‐scale SCP production reactor with substrate fed at the bottom. One environment will be found at the top where $N_{O_{2}}$ is low, risking O_2_ depletion. A second environment will be found at the bottom, where cells have abundant O_2_ and substrate. A third environment will be found within the cooling loop, where cells will face a 15°C fall in temperature, together with O_2_ depletion, and a shock of high shear during their transit through the pump. Mimicking the main features of those three environments will be necessary for the design of downscaled experiments. In follow‐up work, the design of the feed points and the associated modification of the model should be jointly carried out to reflect a realistic production environment in which mixing is incomplete.

### 3.8 Sensitivity Analysis

Several parameters that were kept fixed in all the previous analyses can, in reality, deviate or even vary over time. This section discusses the impacts of using different values for the (i) kinetic and stoichiometric parameters in the model of microorganisms, (ii) fermentation temperature, and (iii) coalescing properties of the fermentation broth.

Industrially, a higher SCP production rate is often sought by increasing the biomass yield. If this yield increased by 10% (from 0.63 to 0.69 g_x_/g_S_, a value still lower than the maximum found and shown in Table A1 in the Appendix A), the production of 1 mole of biomass will require 8.7% and 14.5% less substrate and O_2_, respectively, when D is kept at 0.15 h^−1^. Consequently, the bioreactor productivity ($R_{x}$, in mol_x_/s) increases between 18% and 20% for the whole range of $v_{\mathrm{sG}}^{\mathrm{mean}}$.

At the assumed $K_{S}$ of 5 × 10^−4^ mol/kg_L_, no significant gradients are expected for the substrate concentration within the bioreactor. However, it has been reported that mutations of the SCP production strain *Fusarium graminearum*, growing in continuous fermentations, tend to develop more efficient substrate uptake systems, that is, lower $K_{S}$ (Trinci 1994). If $K_{S}$ was 1 order of magnitude lower than we initially assumed, the average $C_{S}$ and the characteristic time for its consumption would also lower by 1 order of magnitude. Consequently, $\tau_{S}$ will be 1 order of magnitude lower than $\tau_{\mathrm{mix},L}$, thus $C_{S}$ gradients may then be expected. However, that potential problem has a simple solution, feeding the substrate at different heights along the liquid flow pattern.

The values of $q_{S}^{\max}$ and $m_{S}$ do not significantly influence the model results because in the case of the former, $C_{S}$ is lower than $K_{S}$, and in the case of the latter, operating the fermentation at high D values makes the relative requirements of substrate for maintenance irrelevant.

Increasing the temperature is a design choice that has been argued to be beneficial for cooling the bioreactor (Solomons and Litchfield 1983). If the temperature was increased from 30°C to 45°C and the kinetic and stoichiometric parameters in the model of microorganism were kept unaltered, the only results that would change in the model are the mass transfer rates. The value of $k_{L}a$ would increase by 39% while the O_2_ saturation concentrations would decrease by 25%, overall leading to a slight 4% increase in $N_{O_{2}}$ and consequently lowering by 4% the amount of gas that would need to be fed ($F_{G,\mathrm{in}}^{N}$). This would possibly reduce the operation costs. However, the most significant change expected after the temperature increase would be the type of utility employed for cooling the bioreactor. At T = 30°C, chilled water is needed for achieving a 15°C gradient in the cooling agent. At T = 45°C, cooling water may be used instead. According to (Towler and Sinnott 2013), the use of cooling water is between 4 and 8 times cheaper than chilled water per unit of heat removed. An economic analysis of this process will reveal the true benefits of increasing the process temperature.

The presence of coalescence inhibitors in the broth composition will increase the value of $k_{L}a$, improving $N_{O_{2}}$ while keeping the driving force for mass transfer unchanged. For example, adding 5% ethanol to pure water increased the value of $k_{L}a$ up to a factor 6 (Puiman et al. 2022). Using ethanol to control broth coalescence will mean that another nutrient should be limiting, for instance the nitrogen, phosphorus, or sulfur sources. If coalescence inhibitors would increase $k_{L}a$ threefold, a 75% and 115% increase in the O_2_ utilization and biomass concentrations may be expected, respectively. Although increases in $N_{O_{2}}$ are intuitively desirable, this will also make the already significant challenges of the cooling system even more severe. If the cooling system and cells are designed to be compatible with each other, then further increases in $N_{O_{2}}$ will be welcome.

### 3.9 The Overall Picture

The predicted large O_2_ supply rate enables a SCP production rate of 58 kt/y in a 600 m^3^ bubble column. Cells may be produced at a concentration up to 114 g/kg.

However, O_2_ utilization is only 27% at maximum productivity because the energy enabling mass transfer is provided by gas flow. It may be possible to increase O_2_ utilization by (i) implementing lower superficial gas velocities, (ii) increasing the bioreactor height, and (iii) inhibiting the coalescence of gas bubbles in the broth. As consequence, the behavior of the gas phase will be steered toward a plug‐flow behavior. Thus, larger gradients in O_2_ transfer capacity may be found, which is not necessarily a drawback if cells are not affected by it. Other bioreactor designs may be adopted to uncouple the gas flow from the energy driving mass transfer, such as in a stirred tank.

At a dilution rate of 0.15 h^−1^, ethanol may be fed to the bioreactor at a concentration of only 224 g/kg. Higher $C_{S,\mathrm{in}}$ values lead to dissolved O_2_ concentrations that may be too low to support the biomass growth on ethanol. At a lower dilution rate, more ethanol could be supplied while keeping a sufficient concentration of dissolved O_2_. This would increase the $C_{x}$ but, at the same time, it would decrease the yield of biomass on ethanol.

The two factors that have a large potential for becoming constraints in achieving the predicted SCP production capacity, (i) CO_2_ concentration and (ii) heat production, may ultimately, if no other solution is found, be attenuated by limiting the O_2_ supply and sacrificing productivity. Still, how influential the changes in the production rate are over the final economic feasibility of the project, is a question that remains open.

Additional trade‐offs may arise when process economics are considered, which should be discussed along with the technical results obtained in this study to reveal the most optimal configuration. However, economic assessment is outside of the scope of this paper. Similarly, environmental impacts, which are closely related to energy and water use and nutrient washout, should also be considered when determining the optimal operation conditions. Recycling spent centrifugate with nutrients after biomass separation should also be taken into account.

## 4 Conclusions

For a 600 m^3^ bubble column reactor, a model was constructed and used for predicting achievable biomass growth on ethanol. Operating conditions were identified that should allow SCP production of up to 7198 kg/h (58 kt/y on a 330 days‐per‐year basis). The productivity is driven by the maximum rate at which O_2_ can be transferred to the liquid.

The use of pure O_2_ rather than air allows a fourfold increase in productivity. Most of the pure O_2_ remains unconsumed, making its recycling, after CO_2_ capture, an attractive process option. The high O_2_ transfer rate achievable with pure O_2_ (0.24–1.10 mol/[kg_L_ h]) leads to high dissolved CO_2_ concentrations (up to 20 mmol/kg_L_) and large heat loads (139 W/kg). At an industrial scale, such operation might lead to both CO_2_ inhibition and microbial stress within the external cooling loop due to a (i) 15°C temperature drop, (ii) shear at the pump, and (iii) O_2_ depletion, on average once every 7.5 min. These aspects need experimental testing for specific microbial strains.

According to the model calculations, a high biomass concentration can be achieved (114 g/kg) with relatively dilute ethanol feed (224 g/kg). This ethanol will be virtually completely consumed, so the yield of biomass on ethanol is 0.61 g_x_/g_ethanol_. The feeding of ethanol through a single point may lead to (i) ethanol concentration gradients and (ii) possible inhibition, signaling the need for using several feeding points along the liquid circulation path.

The model developed in this study serves as the basis for subsequent techno‐economic analysis and life‐cycle assessment, to determine the most sustainable operating conditions in addition to the technically feasible ones. Furthermore, the model can also be used to identify the microbial strain with the best set of properties. Finally, the concept model is also considered to be adaptable to model the bioreactor conditions and cell growth on other substrates than ethanol.

## Nomenclature

Symbol: Quantity, Unit
*A*
: Area, m^2^
$C_{i}$
: Concentration of compound *i* in liquid, mol_i_/kg_L_
$C_{O_{2}}^{*}$
: Equilibrium concentration of O_2_ in liquid, mol/kg_L_
*c*_*p*_
: Heat capacity, kJ/(kg K)
D
: Dilution rate of the broth (*F*_*L,*out_/*M*_*L*_), 1/h
$D_{i}$
: Film diffusivity of compound *i*, m^2^/s
$D_{R}$
: Diameter of bioreactor, m
$D_{G}$
: Dispersion coefficient for the gas phase, m^2^/s
$D_{L}$
: Dispersion coefficient for the liquid phase, m^2^/s
*F* ^*N*^
: Mole flow rate, mol/h
*F* ^*m*^
: Mass flow rate, kg/h
*H*
: Height, m
$K_{H,i}$
: Henry's coefficient of compound *i*, mol/(kg_L_ bar)
$K_{S}$
: Affinity constant for substrate, mol/m^3^
$k_{L}a$
: Mass transfer coefficient, 1/h
*M*
: Mass, kg
$m_{S}$
: Maintenance substrate requirements, mol_S_/(mol_x_ h)
$N_{i}$
: Transfer rate of compound *i* from gas to liquid, mol_i_/(kg_L_ h)
*p*
: Pressure (absolute), bar
$p_{W}^{\mathrm{sat}}$
: Saturated vapor pressure of water, bar
Q
: Heat, kW
$q_{i}$
: Biomass‐specific production rate of *i*, mol_i_/(mol_x_ h)
$q_{S}^{\max}$
: Maximum substrate consumption rate, mol_S_/(mol_x_ h)
*R*
: Ideal gas constant, J/(mol K)
$R_{i}$
: Production rate of *i*, mol_i_/h
*r*_*i*_
: Mass‐specific production rate of compound *i*, mol_i_/(kg_L_ h)
T
: Temperature, K or °C
*U*
: Heat transfer coefficient, kW/(m^2^ K)
*V*
: Volume, m^3^
v
: Velocity, m/s
$v_{\mathrm{sG}}$
: Superficial gas velocity, m/s
$Y_{X/S}^{\max}$
: Maximum yield of biomass on substrate, g_x_/g_S_
$y_{i}$
: Mole fraction of compound *i* in gas, mol_i_/mol_G_

### Greek Symbols

${∆}_{r}G^{o}$
: Standard Gibbs energy of reaction *r*, kJ/mol
${∆}_{r}H^{o}$
: Standard enthalpy of reaction *r*, kJ/mol
${∆}_{\mathrm{evap}}H$
: Latent heat of water, kJ/mol
$∆T_{\mathrm{lm}}$
: Logarithmic mean temperature difference, K
μ
: Biomass‐specific growth rate, 1/h
τ
: Characteristic time, s

### 1 Subscripts and Superscripts

*Bot*
: At the bottom of the bioreactor
*Cool*
: For cooling
*G*
: For the gas phase
*i*
: For compound *i*
In: Inflow
*L*
: For liquid phase, free liquid plus microbial cells
Mean: Logarithmic mean of top and bottom of the bioreactor
*N,*feed: Liquid feed flow that contains nitrogen source
Out: Outflow
*R*
: Of the bioreactor
*S*
: For substrate
*S,*feed: Liquid feed flow that contains substrate
Top: At the top of the bioreactor
*W*
: For water
*x*
: For dry biomass

## Author Contributions

**E. Almeida Benalcázar:** lead writer, conception, interpretation, data analysis. **W. A. van Winden:** conception, interpretation, reviewing. **L. Puiman:** conception, interpretation, reviewing. **J. A. Posada:** conception, interpretation, reviewing. **M. L. A. Jansen:** conception, interpretation, reviewing. **H. Noorman:** conception, interpretation, reviewing. **A. J. J. Straathof:** conception, interpretation, data analysis, reviewing.

## Conflicts of Interest

The authors declare no conflicts of interest.

## Data Availability

The data that support the findings of this study are available from the corresponding author upon reasonable request. The MATLAB code that generates the output data of this study is available from the corresponding author upon reasonable request.

## 1 A.1. Determination of the Kinetic and Stoichiometric Parameters for the Model of Microorganisms

The values of the kinetic and stoichiometric parameters for the model of microorganisms (Equation 20 and Equation 21) were determined in two ways: (i) from average experimental values reported in the literature (Table A1) when several values were found and (ii) calculated with a thermodynamics‐based method (Heijnen 2013) for parameters with scarce and dispersed literature values. The values for the maximum biomass yield ($Y_{x/S}^{\max}$), the substrate half‐saturation constant ($K_{S}$), and the maximum growth rate ($\mu^{\max}$) were taken from experimental values, while the maintenance coefficient ($m_{S}$) and the maximum substrate uptake rate ($q_{S}^{\max}$) were calculated as explained below.

Using the average of values found in the literature (see Table A1), $Y_{x/S}^{\max}$ = 1.18 mol_x_/mol_S_, $K_{S}$ = 0.001 mol/kg_L_, and $\mu^{\max}$ = 0.22 h^−1^ were obtained.

The value of $m_{S}$ was predicted using Equation A1, which considers that maintenance requires $m_{G}^{0}=\;$4.5 kJ/(mol_x_ h) at 25°C and that the activation energy for the maintenance reactions is $E_{\mathrm{act}}$ = 69 kJ/mol, with R as the ideal gas constant (Heijnen 2013).

(A1)
$$m_{S}=\frac{m_{G}^{0}\text{exp}\left[\frac{-E_{act}}{R}\left(\frac{1}{T^{o}}-\frac{1}{T}\right)\right]}{-{∆}_{r}G_{\mathrm{Cat}}^{0}}$$

To calculate ${∆}_{r}G_{\mathrm{Cat}}^{0}$, which is the standard Gibbs free energy of the catabolic reaction (the electron donor combustion reaction), was conveniently calculated through the dot product between vectors (see Equation A2). The stoichiometric coefficients of the reaction and the standard Gibbs free energies of formation of the substances involved in the reaction were assembled in column vector Cat and row vector ${∆}_{f}G^{0}$, respectively. Bold font is used here to denote vectors and matrices.

(A2)
$${∆}_{r}G_{\mathrm{Cat}}^{0}={∆}_{f}G^{0}\cdot \mathrm{Cat}$$

**Table A1:** Published kinetic parameters for aerobic cell growth on ethanol. Temperatures were between 26°C and 35°C.

$Y_{x/S}^{\max}$ (g_X_/g_S_)	$\mu^{\max}$ (h^‐1^)	$m_{S}$ (g_S_/(g_X_·h))	$K_{S}$ (mmol/kg)	Microorganism^a^	Reference
0.23	0.17			*Methylorubrum extorquens*	(Van Peteghem et al. 2022)
0.28				*Acetobacter pasteurianus*	(Luttik et al. 1997)
0.38	0.048			*Corynebacterium glutamicum*	(Van Peteghem et al. 2022)
0.47	0.10			*Metschnikowia pulcherrima*	(Van Peteghem et al. 2022)
0.47	0.24			*C. glutamicum*	(Arndt et al. 2008)
0.50	0.12		0.43	*S. cerevisiae*	(Mor and Fiechter 1968)
0.58				*Mycobacterium vaccae*	(Blevins and Perry 1971)
0.59	0.13			*S. cerevisiae*	(Taherzadeh et al. 2000)
0.59				*Pseudomonas oxalatus*	(Rutgers et al. 1989)
0.61				*S. cerevisiae*	(Verduyn et al. 1991)
0.61				*S. cerevisiae*	(De Jong‐Gubbels et al. 1995)
0.64				*Rhodotorula* sp. Y‐38	(Yech 1996)
0.68				*Candida boidinii*	(Lücke et al. 1976)
0.68	0.20			*Candida utilis*	(Hernandez and Johnson 1967)
0.69				*C. utilis*	(Verduyn et al. 1991)
0.70	0.125			*S. cerevisiae*	(Paalme et al. 1997)
0.70	0.17			*Wickerhamomyces anomalus*	(Van Peteghem et al. 2022)
0.78		0.11		*Acinetobacter calcoaceticus*	(Abbott et al. 1974)
0.82	0.16			*Cyberlindnera saturnus*	(Van Peteghem et al. 2022)
0.83		0.018		*C. utilis*	(Heijnen and Roels 1981)
0.85	0.96	0.115	1.2	*A. calcoaceticus*	(Preez et al. 1981)

^a^ Current names may differ.

The stoichiometry of the catabolic reaction (Ethanol—3 O_2_ + 2 CO_2_ + 3 H_2_O) led to the vector Cat shown below. The standard Gibbs free energies of formation are shown in Table A2.

(A3)
$$\mathrm{Cat}=\left[\begin{array}{r} -1\\ 0\\ -3\\ 2\\ 3\\ 0 \end{array}\right]\;\begin{array}{c} S\\ x\\ O_{2}\\ CO_{2}\\ H_{2}O\\ NH_{3} \end{array}$$

${∆}_{r}G_{\mathrm{Cat}}^{0}$ = −1319 kJ/mol_S_ led to $m_{S}$ = 0.005 mol_S_/(mol_x_ h), which is in the order of magnitude of published values (see Table A1), regarded acceptable and thus used. To calculate $q_{S}^{\max}$, the Pirt equation at maximum growth rate was used (see Equation A4). That led to $q_{S}^{\max}\;$= −0.19 mol_S_/(mol_x_ h). No literature data of $q_{S}^{\max}$ were found for comparison.

(A4)
$${-q}_{S}^{\max}=\frac{1}{Y_{x/S}^{\max}}\mu^{\max}+m_{S}$$

**Table A2:** Physical properties used for the compounds considered in this study (Heijnen 2013).

Compound	${∆}_{f}G_{i}^{0}$ (kJ/mol_i_)
Ethanol	−181.8
Cells (CH_1.8_O_0.5_N_0.2_)	−67.0
Oxygen	0
Carbon dioxide	−394.4
Water	−237.2
Ammonia	−16.4

## 2 A.2. Determination of the Process Reaction Stoichiometry

The stoichiometry of the process reaction was calculated using an operation between vectors and matrices. The vector Pro contains the stoichiometry of the process reaction with elements located in the same order as in Cat (Equation A3). The stoichiometry of the process reactions is defined from combinations between the catabolic and the anabolic reactions (An). An is a reaction where all the electrons from the substrate are directed to biomass (see Equation A5). Cat and An are first combined into a biomass formation reaction (Bio) where the stoichiometric coefficient of the substrate is $1/Y_{x/S}^{\max}$. The stoichiometry of the other substances involved in Bio is defined by balancing carbon, hydrogen, oxygen, and nitrogen atoms, which is done using an elemental matrix (E). E contains the elemental composition (atoms C, H, O, and N) of the substances involved in the process, see Equation A6 (Noorman et al. 1991). Both Bio and E are split into known and calculated parts, denoted with the subscripts *m* and *c*, respectively. The known parts of E and Bio are those assigned to ethanol and biomass; the calculated parts of E and Pro are thus those assigned to O_2_, CO_2_, water, and NH_3_. The whole matrix E, as well as the different splits of it and of Bio, are shown below. The final vector Bio is assembled by joining its parts, as in Equation A11.

Lastly, the final stoichiometry of Pro is determined using the Pirt relation, where additional contribution of Cat is provided to maintenance (see Equation A12).

(A5)
$$\mathrm{An}=\left[\begin{array}{r} -0.35\\ 1\\ 0\\ -0.3\\ 0.45\\ -0.2 \end{array}\right]\;\begin{array}{c} S\\ x\\ O_{2}\\ CO_{2}\\ H_{2}O\\ NH_{3} \end{array}$$

$$S\;x\;O_{2}\;CO_{2}\;H_{2}O\;NH_{3}$$

(A6)
$$E=\left[\begin{array}{cccccc} 2 & 1 & 0 & 1 & 0 & 0\\ 6 & 1.8 & 0 & 0 & 2 & 3\\ 1 & 0.5 & 2 & 2 & 1 & 0\\ 0 & 0.2 & 0 & 0 & 0 & 1 \end{array}\right]\begin{array}{c} C\\ H\\ O\\ N \end{array}$$

(A7)
$$E_{m}=\left[\begin{array}{cc} 2 & 1\\ 6 & 1.8\\ 1 & 0.5\\ 0 & 0.2 \end{array}\right]$$

(A8)
$$E_{c}=\left[\begin{array}{cccc} \;0\; & \;1\; & \;0\; & \;0\;\\ 0 & 0 & 2 & 3\\ 2 & 2 & 1 & 0\\ 0 & 0 & 0 & 1 \end{array}\right]$$

(A9)
$${\mathrm{Bio}}_{m}=\left[\begin{array}{c} -1/Y_{x/S}^{\max}\\ 1 \end{array}\right]$$

(A10)
$${\mathrm{Bio}}_{c}={E_{c}}^{-1}\times E_{m}\times {\mathrm{Bio}}_{m}$$

(A11)
$$\mathrm{Bio}=\left[\begin{array}{l} {\mathrm{Bio}}_{m}\\ {\mathrm{Bio}}_{c} \end{array}\right]$$

(A12)
$$\mathrm{Pro}=\mathrm{Bio}+\frac{m_{S}}{\mu }\mathrm{Cat}$$

For D=μ=0.15 h^−1^, $Y_{x/S}^{\max}$ = 1.178 mol_x_/mol_S_, the stoichiometry of Pro is:

– 0.88 Ethanol – 1.60 O_2_ – 0.20 NH_3_ + CH_1.8_O_0.5_N_0.2_ + 0.77 CO_2_ + 2.05 H_2_O, or:

$$\mathrm{Pro}=\left[\begin{array}{r} \begin{array}{c} -0.88\\ 1.00 \end{array}\\ -1.60\\ 0.77\\ 2.05\\ -0.20 \end{array}\right]$$

## 3 A.3. Additional Data

**Table A3:** Physical properties at 30°C such as those used in the model.

Property	Value	Source
*c*_*p*_	4.18 kJ/(kg K)	(Perry and Green 2003)
$D_{{\mathrm{CO}}_{2}}$	2.70 × 10^−9^ m^2^/s	(Wilke and Chang 1955)
$D_{O_{2}}$	3.21 × 10^−9^ m^2^/s	(Wilke and Chang 1955)
$K_{H,{\mathrm{CO}}_{2}}$	28.90 mmol/(kg_L_ bar)	(Sander 2015)
$K_{H,O_{2}}$	1.09 mmol/(kg_L_ bar)	(Sander 2015)
${∆}_{\mathrm{evap}}H$	42.3 kJ/mol	(Perry and Green 2003)
$p_{W}^{\mathrm{sat}}$	0.0418 bar	(Perry and Green 2003)

**Table A4:** List of characteristic times.

Mechanism		Equation	Equation	Comments
Mixing	Liquid mixing	$\tau_{\mathrm{mix},L}=1.6{\left(\frac{{D_{R}}^{2}}{gv_{\mathrm{sG}}^{\mathrm{mean}}}\right)}^{\frac{1}{3}}{\left(\frac{H_{L}}{D_{R}\left(1-\varepsilon_{G}\right)}\right)}^{2}$	A13	The calculation of $\tau_{\mathrm{mix},L}$ and $\tau_{\mathrm{mix},G}$ follows the approaches outlined by van't Riet and van der Lans (2011) and Sweere et al. (1987), respectively. Equation A13 is specifically applicable for bubble columns with a height‐over‐diameter ratio higher than 3. Both models consider the circulation of liquid and gas throughout the entire height of the column, including the effect of axial dispersion coefficient $D_{G}$. $H_{L}$ is corrected for the height of the gassed liquid.
	Gas mixing	$\tau_{\mathrm{mix},G}=\frac{{H_{L}}^{2}}{{{\left(1-\varepsilon_{G}\right)}^{2}D}_{G}}$	A14	
		$D_{G}=78{\left(D_{R}v_{\mathrm{sG}}^{\mathrm{mean}}\right)}^{\frac{3}{2}}$	A15	
Mass transfer	O_2_	$\tau_{\mathrm{MT}}=\frac{1}{k_{L}a\left(\frac{C_{O_{2}}^{*}-C_{O_{2}}}{C_{O_{2}}^{*}}\right)}$	A16	$\tau_{\mathrm{MT}}$ is defined as the inverse of the mass transfer coefficient multiplied by the non‐dimensionalized driving force. The driving force is included because O_2_ is not allowed to deplete completely.
Conversion	Substrate	$\tau_{S}=\left|\frac{C_{S}}{q_{S}C_{x}}\right|$	A17	The characteristic times describing the microbial reactions are calculated by dividing the concentration of each of the substances involved by their conversion or production rates.
	O_2_	$\tau_{O_{2}}=\left|\frac{C_{O_{2}}}{q_{O_{2}}C_{x}}\right|$	A18	
	CO_2_	$\tau_{CO_{2}}=\frac{C_{{\mathrm{CO}}_{2}}}{q_{CO_{2}}C_{x}}$	A19	
Transport	Gas flow across bioreactor	$\tau_{F_{G}}=\frac{H_{L}}{1-\varepsilon_{G}}\frac{\varepsilon_{G}}{v_{\mathrm{sG}}^{\mathrm{mean}}}$	A20	$\tau_{F_{G}}$ is calculated considering the gas will flow through the fraction of the column's cross‐sectional area available for the gas only. $H_{L}$ is corrected for the height of the gassed liquid.
Heat	Heat production	$\tau_{\mathrm{Heat}}=\frac{M_{L}∙4.18\frac{J}{g}∙1^{o}C}{Q_{r}}$	A21	$\tau_{\mathrm{Heat}}$ is defined as the time it would take for the fermentation broth to increase its temperature by 1°C, assuming no cooling.

**Table A5:** Overview of input values and conditions at a dilution rate of 0.15 h^−1^.

Variable	Unit	Pure O_2_	Air
$Y_{x/S}^{\max}$	mol_x_/mol_S_	1.18	1.18
	g_x_/g_S_	0.63	0.63
$K_{S}$	mmol/kg	0.50	0.50
$m_{S}$	mol_S_/(mol_x_ h)	0.005	0.005
$q_{S}^{\max}$	mol_S_/(mol_x_ h)	0.19	0.19
*T*	°C	30	30
$p_{\mathrm{top}}$	Bar	1.2	1.2
$y_{O_{2},\mathrm{in}}$	mol_O2_/mol_G_	1.00	0.21
*D*	h^‐1^	0.15	0.15
$V_{R}$	m^3^	600	600
$D_{R}$	m	5.03	5.03
$H_{R}$	m	30.2	30.2
$H_{R}/D_{R}$	—	6	6
$C_{N,\mathrm{in}}$	mol/kg	11.76	11.76
$C_{N}$	mol/kg	0.059	0.059
$C_{O_{2}}$	mmol/kg	0.069	0.069

**Table A6:** Overview of output values at a dilution rate of 0.15 h^−1^ and the two extreme values of $v_{\mathrm{sG}}^{\mathrm{mean}}$, 0.04 and 0.30 m/s.

Variable	Unit	Pure O_2_		Air	
		Mean superficial gas velocity			
		0.04 m/s	0.30 m/s	0.04 m/s	0.30 m/s
$Y_{x/S}$	mol_x_/mol_S_	1.14	1.14	1.14	1.14
	g_x_/g_S_	0.61	0.61	0.61	0.61
$q_{S}$	mol_S_/(mol_x_ h)	−0.132	−0.132	−0.132	−0.132
$V_{L}$	m^3^	534	423	534	423
$H_{L}$	m	26.9	21.3	26.9	21.3
$M_{L}$	t	534	423	534	423
$p_{\mathrm{bot}}$	bar	3.8	3.3	3.8	3.3
$F_{G,\mathrm{in}}^{N}$	mol/s	74	482	64	448
$y_{O_{2},\mathrm{in}}$	—	1.00	1.00	0.21	0.21
$y_{N_{2},\mathrm{in}}$	—	0.00	0.00	0.79	0.79
$F_{G,\mathrm{out}}^{N}$	mol/s	58	429	64	453
$y_{O_{2},\mathrm{out}}$	—	0.67	0.82	0.13	0.16
$y_{N_{2},\mathrm{out}}$	—	0.00	0.00	0.80	0.78
$y_{{\mathrm{CO}}_{2},\mathrm{out}}$	—	0.29	0.14	0.04	0.02
$y_{W,\mathrm{out}}$	—	0.03	0.03	0.03	0.03
$F_{S,\mathrm{feed}}^{M}$	kg/h	76,945	53,019	79,286	61,436
$C_{S,\mathrm{in}}$	g/kg	42.34	223.67	6.36	31.87
$F_{N,\mathrm{feed}}^{M}$	kg/h	1766	5291	610	1137
$F_{L,\mathrm{out}}^{M}$	kg/h	80,111	63,415	80,111	63,415
$C_{x}$	mol/kg	1.00	4.61	0.15	0.76
	g/kg	24.7	113.5	3.8	18.7
$C_{S}$	mmol/kg	1.1	1.1	1.1	1.1
$C_{CO_{2}}$	mmol/kg	20.1	9.6	2.8	1.5
$N_{O_{2}}$	mol/(kg h)	0.24	1.10	0.04	0.18
$R_{S}$	kmol/h	−71	−258	−11	−42
$R_{x}$	kmol/h	80	293	12	48
	kg/h	1976	7198	304	1187
	kt/y	16	58	2	9
$R_{O_{2}}$	kmol/h	−128	−466	−20	−77
$R_{CO_{2}}$	kmol/h	61	223	9	37
$R_{W}$	kmol/h	164	598	25	99
$R_{N}$	kmol/h	−16	−59	−2	−10
$Q_{r}$	MW	16.3	59.5	2.5	9.8
${∆}_{\mathrm{evap}}H$	MW	0.08	0.63	0.09	0.67
$Q_{\mathrm{cool}}$	MW	16.3	58.9	2.4	9.1
	kW/t	30	139	5	22
$F_{\mathrm{cool}}^{M}$	t/h	933	3382	139	525
$A_{\mathrm{cool}}$	m^2^	774	2805	115	436
$v_{\mathrm{sG}}^{\mathrm{top}}$	m/s	0.06	0.45	0.07	0.48
$v_{\mathrm{sG}}^{\mathrm{bot}}$	m/s	0.02	0.19	0.02	0.17
$\varepsilon_{G}$	—	0.06	0.26	0.06	0.26
${\left[k_{L}a\right]}_{O_{2}}$	h^−1^	150	617	150	617
${\left[k_{L}a\right]}_{{\mathrm{CO}}_{2}}$	h^−1^	138	565	138	565
Energy input by gas sparging	kW/m^3^	392	2940	392	2940
$v_{L}$	m/s	1.13	2.21	1.13	2.21
$D_{L}$	m^2^/s	2.08	4.08	2.08	4.08
$D_{G}$	m^2^/s	7.05	144.6	7.0	144.6
$\tau_{\mathrm{mix},L}$	s	208.6	106.5	208.6	106.5
$\tau_{\mathrm{mix},G}$	s	117.2	5.7	117.1	5.7
$\tau_{\mathrm{MT}}$	s	25.0	6.1	30.7	7.2
$\tau_{S}$	s	30.3	6.6	197.2	39.9
$\tau_{O_{2}}$	s	1.0	0.2	6.7	1.4
$\tau_{CO_{2}}$	s	321.8	34.2	290.8	32.4
$\tau_{F_{G}}$	s	45.2	24.7	45.2	24.7
$\tau_{\mathrm{Heat}}$	s	137.5	30.0	924.7	193.2
